# Intimate partner violence trajectories over a 1-year period in a population-based cohort of women in Kenya: associations with individual and community normative factors

**DOI:** 10.1136/bmjgh-2025-021078

**Published:** 2025-12-25

**Authors:** Michele R Decker, Anaise Williams, Shannon N Wood, Mary Thiongo, Grace Wamue-Ngare, Peter Gichangi

**Affiliations:** 1 Johns Hopkins Center for Global Women's Health and Gender Equity, Baltimore, Maryland, USA; 2 Department of Population, Family, and Reproductive Health, Johns Hopkins Bloomberg School of Public Health, Baltimore, Maryland, USA; 3 International Centre for Reproductive Health—Kenya (ICRHK), Mombasa, Kenya; 4 Department of Sociology, Gender and Development Studies, Kenyatta University, Nairobi, Kenya; 5 Technical University of Mombasa, Mombasa, Mombasa County, Kenya; 6 Department of Public Health and Primary Care, Ghent University, Ghent, Flanders, Belgium

**Keywords:** Africa, Gender-Based Violence, Global Health

## Abstract

**Introduction:**

The global evidence base on intimate partner violence (IPV) prevalence and risk factors is predominantly cross-sectional, limiting clarity in changes over time. Prospective cohort data can inform trajectories, as is needed to guide prevention efforts.

**Aims:**

This study characterises (1) the prevalence of past-year IPV and trajectories among a cohort at two time points spanning 1 year and (2) individual and household factors associated with physical and/or sexual IPV, and IPV trajectories, adjusting for and stratifying on community-level norms that endorse IPV justification.

**Methods:**

Existing cohorts of women aged 15–49 were surveyed in November–December 2020 and November–December 2021 (n=3082_2020_; n=2887_2021_). The analytic sample restricts to married or partner-cohabiting women with complete IPV data at both time points (n=2499). County-level IPV justification norms were computed from Kenya 2022 Demographic and Health Survey data. Descriptive and multivariable regression characterise IPV prevalence, trajectories and help-seeking over 12 months, and associations of individual, dyad and community-normative factors on any IPV and trajectories.

**Results:**

From 2020 to 2021, the prevalence of any past-year physical and/or sexual IPV increased slightly from 11.4% to 12.5%. Over the 12-month period, 8% of women had IPV newly begun, 4% had sustained IPV at both time points, 7% had IPV resolved by 2021 and 81% were IPV-free across time points. Norms that justify physical IPV at the county level influenced associations with trajectories and risk. Generally, individual-level factors were more associated with IPV in settings where IPV justification was less normative. Help-seeking was not associated with IPV cessation.

**Conclusion:**

Overall IPV prevalence persisted over a 1-year period, with dynamic movement in and out of safety, illustrating the unmet needs for prevention and response. Social norms appear to ‘activate’ individual and household IPV risk factors, affirming the need to address the norms that perpetuate IPV.

**WHAT IS ALREADY KNOWN ON THIS TOPIC**

**WHAT THIS STUDY ADDS**

- This study identifies IPV persistence across a 1-year period from 2020 to 2021, with about equal percentages of women moving in and out of safety. Most individual factors, including IPV help-seeking, were not associated with change in IPV across the 1-year period. Generally, individual characteristics were less associated with IPV in places with stronger normative IPV justification.

**HOW THIS STUDY MIGHT AFFECT RESEARCH, PRACTICE OR POLICY**

- This first prospective study of IPV trajectories among adult women in Kenya demonstrates concerning persistence of IPV, highlighting the unmet needs for both primary and secondary prevention. The social normative environment remains an important target for change to enhance women’s safety.

## Background

 Gender-based violence (GBV) threatens health and human rights, with most severe consequences including injury and death.^1 2^ Globally, one in three women experiences physical and/or sexual violence by a partner or sexual violence by a non-partner in their lifetime, and intimate partner violence (IPV) is responsible for over a third of women’s homicides.^3 4^ Accordingly, eliminating GBV features among the United Nations’ Sustainable Development Goals, alongside solutions to achieve gender equality.^5^ Crises and their aftermath increase the risk for GBV, while undermining women’s economic and social standing.^6^,^8^ The COVID-19 pandemic raised concerns for escalated GBV due to economic disruption and subsequent household/relationship stressors, together with social and travel restrictions.^8^,^10^ GBV has increased since COVID-19 in many settings, likely reflecting limited mobility, social isolation, increased time in the home with potential abusers and financial and social stress that support conflict. Simultaneously, mobility restrictions, lack of privacy and fears of transmission can create new barriers to violence-related help-seeking.^9 11^

Longitudinal research on GBV trajectories is extremely limited. Recent evidence from young women in Nairobi illustrates relative consistency in IPV prevalence over an 18-month period that included the COVID-19 pandemic, with young women moving in and out of relative safety.^12^ Researchers know little about the demographic associations with violence cessation among survivors compared with continued partner violence among survivors; however, it is widely understood that the strongest predictor of future victimisation is previous victimisation.^13^ Understanding risk factors for different trajectories is critical to the field: revictimisation is related to a higher risk of depression, anxiety, post-traumatic stress disorder, among other health concerns.^14^ Trajectory data are essential to understand the persistence of IPV over time at the individual level, identify the risks associated with new IPV and estimate how individuals experiencing IPV transition into safety; together, these data can inform and refine IPV prevention and response interventions.

Promoting help-seeking and safe spaces for survivors to disclose abuse and obtain psychosocial or medical support is a key part of preventing harmful IPV trajectories.^15^,^17^ Seeking useful help can reduce post-traumatic stress, self-blame and revictimisation.^17^,^21^ Yet, globally, most women who experience IPV do not seek help, with little meaningful change in help-seeking since low help-seeking was first established by WHO in 2005.^22 23^ Social norms that justify and normalise IPV are understood to be a key contextual factor affecting IPV help-seeking, and therefore revictimisation, as characterised by Heise’s ecological model of gender-based violence.^24^,^26^ For example, a study in Tanzania found that women who wanted to seek help for IPV were often blocked from formal and informal help-seeking due to prevailing societal norms.^27^ Norms that tolerate IPV can also lead to internalised blame or justification of the experience, leading to low rates of seeking help among survivors.^28^ Research on the drivers of IPV and other forms of violence against women has overemphasised individual risk factors such as age, socioeconomic status and exposure to familial abuse, with little current research on how the normative environment impacts IPV trajectories.^29^,^32^ Further, recent work emphasises the importance of evaluating the dyadic relationship between partners in IPV risk analysis: how partners communicate, the division of labour, patriarchal triggers and partner perceptions of not meeting gendered expectations, all of which are affected by the normative environment^33^ and in turn can shape immediate IPV risk. Current thinking in line with socioecological frameworks argues for consideration of higher-order factors at both the relational and normative landscape.^30 34 35^

In Kenya, as in many settings, IPV is prevalent, with an estimated 18.6% of ever-partnered women reporting past 12-month physical and/or sexual IPV on the 2022 Demographic and Health Survey (DHS).^36^ The pandemic appears to have exacerbated GBV in Kenya; women report feeling less safe at home since the start of the pandemic.^37^ In urban Kenya, increases in household tension and conflict were reported during the COVID-19 period, as were modest increases in household violence and increases in violence outside the home; perpetrator(s) were not specified.^38^ In rural Kenya, the risk of domestic violence was variable but statistically stable over 11 weeks following the onset of COVID-19 mitigation restrictions.^39^ More recent research illustrates declines in relationship quality due to COVID-19 restrictions, which elevate IPV risk.^40^ Little is known about the trajectories of IPV among partnered women in Kenya, particularly across the COVID-19 era.

Against this backdrop, this study examines (1) the IPV prevalence and intensity at two time points; (2) contact (physical and/or sexual) IPV trajectories over a 12-month follow-up characterised as sustained safety, IPV cessation, new IPV and sustained IPV and (3) associations of individual and dyad factors on any IPV and IPV trajectories in a cohort of married or cohabiting women in Kenya, adjusting for and stratifying on community-level norms that endorse physical IPV justification. Results provide critical new prospective evidence to guide Kenya’s investments in safety planning and supports for IPV survivors, and insight into the needs of this population in future emergencies and recovery.

## Methods

### Study design and sample

This longitudinal analysis draws on prospective data from two rounds of Performance Monitoring for Action (PMA) Kenya surveys, implemented in November–December 2020 (n=10 008) and November–December 2021 (n=10 857; 12.7% loss-to-follow-up between rounds).^41 42^ PMA Kenya uses a multistage cluster design with probability proportional to size sampling of enumeration areas. Data are collected from 11 counties and weighted for generalisability at the national level. Surveys are conducted in person by locally trained female enumerators, trained in ethical protections on violence against women. All study procedures, survey methodology and data for the PMA study are available at www.pmadata.org.

Eligible study participants include females aged 15–49 within selected households, who provided informed written consent and consent for follow-up, and were selected for participation in a GBV module. The GBV module was limited to married women or women living with a partner, one per household, who could ensure privacy for interview (n=3082, 2020; n=2887, 2021; n=2536 at both time points). The analytic sample was restricted to women with no missing values for IPV at either time point (n=2499). Missingness across covariates was <5%; sample size floats to accommodate. The GBV module was situated at the end of the survey and, within eight questions, covered partner violence (emotional, physical, sexual; items described below), frequency, frequency since COVID-19, violence experienced that was perpetrated by a household member who is not a partner and help-seeking. GBV measures and ethical protections align with best practices for violence-related research.

The DHS data for Kenya 2022 contributed normative data that were aggregated into county-level measures, matched to the PMA survey data covering 11 counties.^36^ The DHS surveys both men and women and is representative at the national, urban/rural and regional levels.^43^ More information on DHS data and sampling design can be found at dhs.org.

### Measures

#### Intimate partner violence

IPV measures used behavioural assessment per best practice.^44^ Specifically, at each time point, women were asked if in the last 12 months their husband/partner (1) insulted, yelled at, screamed or made humiliating remarks; (2) slapped, hit or physically hurt you; (3) threatened with a weapon or attempted to strangle or kill you; (4) pressured or insisted on having sex when you did not want to (without physical force) and (5) physically forced you to have sex when you did not want to. These measures informed characterisation of emotional IPV ((1)); physical IPV ((2) or (3)); sexual IPV ((4) or (5)); contact IPV ((2), (3), (4) or (5)); less severe contact IPV ((2) or (4) and ‘no’ to (3) or (5)) and more severe contact IPV ((3) or (5)). Emotional, physical and sexual IPV categories were not mutually exclusive; less severe contact IPV and more severe contact IPV categories were mutually exclusive.

#### Contact IPV trajectories

Mutually exclusive IPV trajectories were characterised based on contact IPV (sexual and/or physical IPV) experience in 2020 and 2021; specifically, a categorical variable was created to reflect sustained safety (no contact IPV at either time point); IPV cessation (contact IPV in 2020 but no contact IPV in 2021); new IPV (no contact IPV in 2020 and any contact IPV in 2021) and sustained IPV (contact IPV at both time points). For multivariable analysis, binary measures were constructed of new IPV versus sustained safety and sustained IPV versus IPV cessation.

#### Contact IPV help-seeking

At each time point, help-seeking for contact IPV was coded as ‘1’ if the respondent reported contact IPV and responded ‘yes’ to “thinking about the experiences of relationship conflict we have just discussed, have you tried to seek help in the last 12 months?” Formal help-seeking was coded as ‘1’ if the respondent sought help and reported seeking help from either a lawyer, medical personnel, police, social service organisation or violence support programme or hotline. Informal help-seeking was coded as ‘1’ if the respondent sought help and reported seeking help from either own family, partner’s family, current or former partner, friend, neighbour or religious leader; help-seeking categories were not mutually exclusive.

#### Independent variables

Independent variables were based on the 2020 survey unless noted otherwise. Sociodemographic characteristics included age (15–29, 30–39, 40–49), education (primary or lower vs secondary or higher), parity (0–2, 3+), lives with extended family (yes/no) and rurality (rural/urban). Economic measures included household wealth (low, middle, high; self-reported), savings (yes/no), land ownership (yes/no), mobile money account ownership (yes/no), income loss due to COVID-19 (yes/no) and past year paid work (yes/no). Partner dyad characteristics included partner cohabitation (yes/no), partner education (primary or lower vs secondary or higher) and other wives (yes/no). Decision-making measures included whether respondent participates in decisions on her own earnings (yes/no or no earnings) and decisions on her husband’s earnings (yes/no).

Two categorical measures of change in independent variables across the two time points were constructed. The first was for paid work (1=continuous non-work, 2=worked to not work, 3=not worked to worked and 4=continuous work). The second was for decision-making participation across the two decision-making measures (1=no decision-making participation at either time point, 2=decrease in the number of decisions participated in, 3=increase in the number of decisions participated in, 4=participated in the same number of decisions at both time points, among those with participation in at least one of the two decisions).

#### County-level norms that justify IPV (from DHS)

A proxy for county-level injunctive norms that justify IPV was created based on responses to five scenarios within the 2022 Kenya DHS. Participants were asked to indicate whether IPV was justifiable (yes/no) if (1) wife goes out without telling husband, (2) wife neglects the children, (3) wife argues with husband, (4) wife refuses to have sex with husband and (5) wife burns food. By gender, an individual-level count variable was constructed to sum the number of items the respondent replies ‘yes, justified’ (0–5). The intraclass correlation coefficient and dispersion of the individual-level count measure, by gender, were explored across counties graphically to assess county-level clustering. Using individual-level DHS survey weights, county-level measures for women and men were constructed by aggregating the average individual-level count variable at the county level. The gender-specific DHS county-level aggregate norms measure (0–5) was merged into the PMA survey dataset. Using the mean of the county-level measure, counties were categorised as either low (at or below average) or high (above average) on norms that justify IPV.

### Statistical analysis

The weighted proportions of past-year any IPV, emotional IPV, physical IPV, sexual IPV, contact IPV, less severe contact IPV and severe contact IPV (defined above), as well as the prevalence of help-seeking, were explored at both time points. The pattern of women moving in and out of safety from 2020 to 2021, for contact IPV overall, as well as less severe contact IPV and severe contact IPV, was visualised in a Sankey diagram. Independent variables were taken from data collected at the first time point, unless noted otherwise, and explored across the full sample. Bivariate analysis to examine the prevalence of independent variables by any contact IPV and IPV trajectory was conducted; differences in independent variables by any contact IPV and IPV trajectory were assessed via design-based F-statistics. County-level IPV prevalence and average county-level norms that endorse IPV justification, by gender, were displayed in a bar graph by county.

Backwards stepwise logistic regression was used to identify multivariable specifications for three binary outcome variables: (1) any contact IPV versus no contact IPV, (2) new contact IPV versus sustained safety and (3) sustained IPV versus IPV cessation. Independent variables excluding help-seeking with bivariate associations p≤0.2 that differed by any IPV for (1) and IPV trajectories for (2) and (3), plus age, were included in the backwards stepwise regressions. Starting with all relevant independent variables, the variable with least significance in the model was removed and model fit assessed after each removal. Due to the use of STATA’s ‘SurveySet’ to account for complex sampling design, the AIC (Akaike Information Criterion)/BIC (Bayesian Information Criterion) were not able to be generated to assess model fit. As an alternative, we used the Hosmer-Lemeshow test in which a higher p value/less significant F-statistic suggests better fit.^45^ For the model of best fit for each of the three outcomes, specifications were presented (1) overall without IPV justification norms, (2) overall adjusting for both men’s and women’s IPV justification norms, (3) among counties with less IPV justification norms measured among women, (4) among counties with more IPV justification norms measured among women, (5) among counties with less IPV justification norms measured among men and (6) among counties with more IPV justification norms measured among men. All analyses were conducted using STATA 17.0 (College Station, Texas). All analyses are weighted to account for survey design.

Procedures followed best practices for violence research. Prior to survey implementation, enumerators received GBV-specific training on confidentiality and privacy, asking questions in a non-judgemental manner, monitoring for emotional upset and referring to support services. Privacy checks embedded within ODK (Open Data Kit) software ensured that women completed sensitive questions in a private space. In line with best practices for violence research, all participants were given resource information, inclusive of GBV supports, reproductive health and COVID-related resources, regardless of violence disclosure.^44 46^

### Patient and public involvement

It was not appropriate or possible to involve patients or the public in the immediate design, conduct, reporting or dissemination plans, though extensive community engagement informed the motivation for the study, including the inclusion of the normative environment measures.

## Results

**Figure 1:**
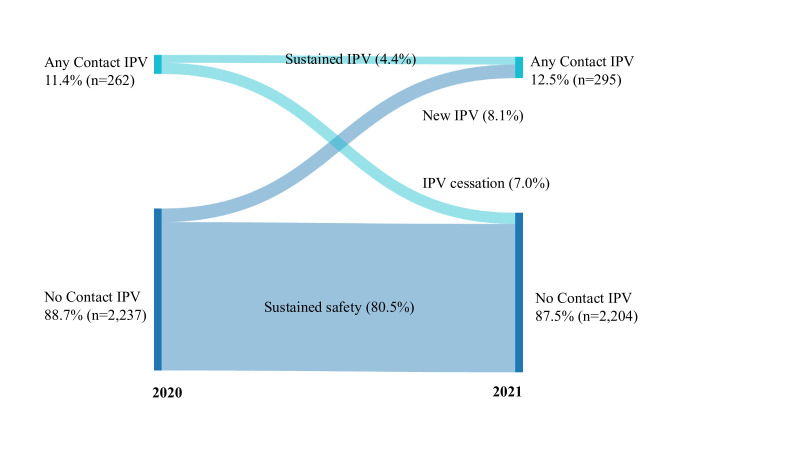
Trajectories of past-year contact intimate partner violence (IPV) from 2020 to 2021 (n=2499, weighted)

Overall, 21.5% and 24.4% of women reported any past-year physical, sexual or emotional IPV in 2020 and 2021, respectively (table 1). Contact IPV prevalence was 11.4% in 2020 and 12.5% in 2021 (table 1, figure 1). In 2020, 5.3% of women experienced less severe contact IPV only and 6.1% experienced severe contact IPV; in 2021, 7.1% of women experienced less severe contact IPV only and 5.4% experienced severe contact IPV (table 1, online supplemental figure 1). Less than half of the contact IPV survivors sought help from any source at both time points, with only 7.5% and 4.4% in 2020 and 2021, respectively, seeking formal help.

**Table 1:** Prevalence of past-year IPV and related help-seeking in 2020 and 2021, weighted

	2020	2021
	%	
*Among full sample*	n=2499*	n=2499*
Any past-year IPV	21.5	24.4
Physical†	7.1	7.7
Sexual†	6.8	7.7
Emotional†	19.1	20.7
Contact IPV^±^	11.4	12.5
Less severe contact IPV^+^	5.3	7.1
Severe contact IPV^~^	6.1	5.4
*Among those with contact IPV*	n=262	n=295
Help-seeking in the past 12 months	44.3	37.3
Formal‡	7.5	4.4
Informal§	40.8	34.5

* Among married women or women currently living with a man with no missing values at both time points for IPV variables.

† Not mutually exclusive.

‡ Formal help-seeking includes sought lawyer, medical provider, police, social organisation, or hotline

§ Informal help-seeking includes sought boyfriend, family, friend, husband, in-law, neighbour or religious leader.

IPV, intimate partner violence.

Across the full sample at the first wave of data collection, less than half (40.5%) had secondary or higher education, and most (74.3%) lived in rural areas and had three or more children (64.1%). Slightly over half (57.4%) reported participating in paid work in the past 12 months. Most (77.1%) reported experiencing household income loss due to COVID-19. Any participation in decisions on how to spend own earnings and decisions on how to spend husband’s earnings was 52.8% and 55.1%, respectively. In bivariate analysis, any contact IPV at either time point was positively associated with respondent lower education (p<0.01), having more children (p<0.01), lower household wealth (p<0.01), no savings (p<0.01), no mobile money account membership (p=0.02), lower partner education (p<0.01), husband having other wives (p<0.01) and not participating in decisions on husband’s earnings (p<0.01) (table 2).

**Table 2:** Sample characteristics, overall and by physical and/or sexual (contact) IPV at either time point (n=2499*), weighted

	Overall 100% (n=2499)*	Any contact IPV (2020 or 2021) 19.5% (n=487)	No contact IPV (2020 and 2021) 80.5% (n=2012)	P value†
		**Row %**	**Row %**	
*Sociodemographic measures*				
Age group (years)				0.81
15–29	38.5	19.5	80.5	
30–39	41.4	18.9	81.1	
40–49	20.1	20.6	79.4	
Education				**<0.01**
Primary or below	59.5	22.5	77.5	
Secondary or higher	40.5	15.0	85.0	
Parity				**<0.01**
0–2 children	35.9	15.5	84.5	
3+ children	64.1	21.7	78.3	
Lives with extended family				0.70
No	80.7	19.3	80.7	
Yes	19.3	20.1	79.9	
Residence				0.40
Urban	25.7	17.8	82.2	
Rural	74.3	20.1	79.9	
*Economic measures*				
Wealth				**<0.01**
Lowest	40.2	24.4	75.6	
Middle	33.9	17.5	82.5	
Highest	25.9	14.4	85.6	
Any savings				**<0.01**
No	50.6	23.1	76.9	
Yes	49.4	15.8	84.2	
Owns land alone or jointly				0.85
No	63.9	19.6	80.4	
Yes	36.1	19.2	80.8	
Paid work in the past 12 months				0.36
No	42.6	18.5	81.5	
Yes	57.4	20.2	79.8	
Mobile money account ownership				**0.02**
No	23.7	24.7	75.3	
Yes	76.3	17.9	82.1	
Income loss due to COVID-19 restrictions				0.65
No	22.9	20.4	79.6	
Yes	77.1	19.2	80.8	
*Partner dyad*				
Living with partner/nuclear family				0.84
No	19.6	19.8	80.2	
Yes	80.4	19.4	80.6	
Partner education				**<0.01**
Primary or below	51.2	22.5	77.5	
Secondary or higher	48.8	16.3	83.7	
Husband has other wives				**<0.01**
No	88.6	18.1	81.9	
Yes	11.4	30.3	69.7	
*Household spending decision-making*				
Participates: own earnings				0.62
No	47.2	19.0	81.0	
Yes	52.8	20.0	80.0	
Participates: husband’s earnings				**<0.01**
No	44.9	24.1	75.9	
Yes	55.1	15.7	84.3	

Measures taken at first time point (2020).

* Among married or cohabiting women with no missing values for the GBV module at both time points.

† P value of the design-based F-statistic by IPV status; bold indicates p≤0.20.

GBV, gender-based violence; IPV, intimate partner violence.

Across the sample, 80.5% of women experienced sustained safety (no IPV) across time points, 8.1% of women experienced new contact IPV in 2021, 4.4% had sustained contact IPV at both time points and 7.0% had IPV cessation in 2021 (figure 1). Independent variables that significantly varied across these four IPV trajectories were primary education (p<0.01), three or more children (p=0.02), household wealth (p<0.01), any savings (p<0.01), mobile money account ownership (p<0.01), partner education (p<0.01), husband having other wives (p<0.01) and participation in decisions on husband’s earnings (p<0.01) (table 3). Independent variables that were marginally significantly variable across IPV trajectories were paid work trajectory (p=0.17), household income loss due to COVID-19 restrictions (p=0.11) and decision-making participation trajectory (p=0.11). Help-seeking in 2020, overall and by formal status, was not associated with sustained IPV compared with IPV cessation (table 3). While any help-seeking in 2021 was not associated with new versus sustained IPV, those who sought specifically formal help-seeking in 2021 were marginally more likely to be women with sustained IPV than women who had new IPV in 2021 (p=0.10) (table 3).

**Table 3:** Sample characteristics bivariate associations with physical and/or sexual (contact) IPV trajectories (n=2499*), weighted

	IPV trajectories				
	Sustained safety 80.5% (n=2012)	IPV cessation 7.0% (n=175)	New IPV 8.1% (n=203)	Sustained IPV 4.4% (n=109)	P value†
	Row %	Row %	Row %	Row %	
*Sociodemographic measures*					
Age group (years)					0.78
15–29	80.5	7.5	7.4	4.6	
30–39	81.1	5.9	8.9	4.1	
40–49	79.4	8.2	7.8	4.6	
Education					**<0.01**
Primary or lower	77.5	7.7	9.0	5.9	
Secondary or higher	85.0	6.0	6.9	2.1	
Parity					**0.02**
0–2 children	84.5	5.3	7.1	3.1	
3+children	78.3	8.0	8.7	5.1	
Lives with extended family					0.71
No	80.7	6.7	8.3	4.3	
Yes	79.9	8.1	7.4	4.6	
Residence					0.43
Urban	82.2	7.5	7.3	3.0	
Rural	79.9	6.8	8.4	4.8	
*Economic measures*					
Wealth					**<0.01**
Lowest	75.6	8.5	9.5	6.4	
Middle	82.5	5.6	7.8	4.1	
Highest	85.6	6.5	6.4	1.5	
Any savings					**<0.01**
No	76.9	8.6	8.4	6.0	
Yes	84.2	5.3	7.8	2.6	
Owns land alone or jointly					0.57
No	80.4	6.9	8.7	4.0	
Yes	80.8	7.1	7.1	5.0	
Paid work 2020–2021					**0.17**
Continuous non-work	83.7	5.4	7.2	3.7	
Worked to not work	76.8	11.3	5.9	5.9	
Not worked to work	78.0	7.4	9.7	4.9	
Continuous work	80.5	6.7	8.6	4.2	
Mobile money account ownership					**<0.01**
No	75.3	9.8	7.5	7.4	
Yes	82.1	6.1	8.3	3.4	
Income loss due to COVID-19 restrictions					**0.11**
No	79.6	5.9	7.7	6.8	
Yes	80.8	7.3	8.2	3.6	
*Partner dyad*					
Living with partner/nuclear family					0.74
No	80.2	8.0	7.3	4.6	
Yes	80.6	6.7	8.3	4.3	
Partner education					**<0.01**
Primary or lower	77.5	7.8	8.9	5.8	
Secondary or higher	83.7	6.1	7.3	2.8	
Husband has other wives					**<0.01**
No	81.9	6.4	7.8	3.9	
Yes	69.7	11.6	10.4	8.3	
*Household spending decision-making*					
Participates: own earnings					0.92
No	81.0	6.9	7.8	4.4	
Yes	80.0	7.1	8.6	4.3	
Participates: husband’s earnings					**<0.01**
No	75.9	8.8	8.8	6.5	
Yes	84.3	5.5	7.5	2.6	
*Decision-making 2020–2021*					**0.11**
Continuous non-participation	74.1	6.6	10.7	8.5	
Reduced	79.7	8.4	8.1	3.9	
Increased	80.0	7.6	8.2	4.2	
Continuous participation	83.2	6.0	7.7	3.1	
Help-seeking for contact IPV (2020)					
No help-seeking	n/a	60.1	n/a	39.9	0.69‡
Any help-seeking	n/a	63.4	n/a	36.6	0.69‡
Any formal help-seeking	n/a	55.8	n/a	44.2	0.71‡
Any informal help-seeking	n/a	65.3	n/a	34.7	0.39‡
Help-seeking for contact IPV (2021)					
No help-seeking	n/a	n/a	66.4	33.6	0.62§
Any help-seeking	n/a	n/a	62.9	37.1	0.62§
Any formal help-seeking	n/a	n/a	40.4	59.6	**0.10§**
Any informal help-seeking	n/a	n/a	65.9	34.1	0.85§

Covariate measures taken at first time point, 2020, unless otherwise noted.

* Among married or cohabiting women with no missing values for the GBV module at both time points.

† P value of the design-based F-statistic across contact IPV trajectories; bold indicates p≤0.2.

‡ Between sustained and cessation.

§ Between new and sustained.

GBV, gender-based violence; IPV, intimate partner violence; n/a, not available.

Across the 11 counties, West Pokot demonstrated the highest norms justification for physical IPV (indicator of IPV tolerance) among both men and women (online supplemental figure 2). For men only, Kilifi and Nyamira had the same level of normative IPV justification as West Pokot (online supplemental figure 2). The intraclass correlation coefficient of the individual-level count measure across Kenya counties was high (>0.10), suggesting normative clustering and differences across counties on the summed IPV normative justification measures.

Multivariable regression results for any contact IPV among the full sample, not adjusting for norms, identified significantly greater odds of any contact IPV among women whose partner has other wives (adjusted OR (aOR) 1.69, 95% CI 1.13 to 2.51) (table 4). Protective factors for any contact IPV included secondary education (aOR 0.78, 95% CI 0.59 to 1.02) and participating in decisions on husband’s earnings (aOR 0.62, 95% CI 0.47 to 0.82). County-level IPV justification norms among women were positively associated with contact IPV (aOR 1.65, 95% CI 1.03 to 2.66), while men’s norms were not associated (table 4). In models stratified by IPV justification norms, most observed associations remained significant in counties with lower normative justification of IPV, but attenuated in counties with stronger normative IPV justification (table 5).

**Table 4:** Multivariable regression of select indicators on any IPV and IPV trajectories

	Unadjusted for norms		Adjusted for norms	
	aOR	95% CI	aOR	95% CI
*Any IPV vs sustained safety*				
Secondary or higher education	0.78*	**0.59 to 1.02**	0.77*	**0.58 to 1.02**
Lowest wealth	Ref		Ref	
Middle wealth	0.75*	**0.55 to 1.02**	0.82	0.60 to 1.11
Highest wealth	0.68*	**0.44 to 1.05**	0.77	0.49 to 1.21
Any savings	0.71**	**0.52 to 0.99**	0.72**	**0.52 to 0.99**
Partner has other wives	1.69**	**1.13 to 2.51**	1.52**	**1.02 to 2.28**
Participates in decisions on husband’s earnings	0.62***	**0.47 to 0.82**	0.63***	**0.47 to 0.83**
More IPV justification county norms (women)	–		1.65**	**1.03 to 2.66**
More IPV justification county norms (men)	–		0.64	0.31 to 1.33
Observations	2493		2493	
*Sustained IPV vs IPV cessation*				
Age 15–29 years	Ref		Ref	
Age 30–39 years	1.3	0.60 to 2.82	1.35	0.63 to 2.88
Age 40–49 years	1.1	0.43 to 2.79	1.13	0.46 to 2.77
Secondary or higher education	0.47	0.18 to 1.18	0.44*	**0.18 to 1.09**
Three or more children (vs 0–2)	1.3	0.60 to 2.82	1.35	0.63 to 2.88
Lowest wealth	Ref		Ref	
Middle wealth	1.33	0.70 to 2.54	1.27	0.65 to 2.48
Highest wealth	0.43	0.15 to 1.26	0.40*	**0.14 to 1.17**
Any savings	0.7	0.30 to 1.65	0.66	0.29 to 1.50
Continuous non-work 2020–2021	Ref		Ref	
Worked to not work 2020–2021	0.95	0.29 to 3.06	0.94	0.31 to 2.89
Not worked to work 2020–2021	1.08	0.36 to 3.27	1.14	0.37 to 3.48
Continuous work 2020–2021	1.41	0.60 to 3.34	1.44	0.61 to 3.39
Mobile money account ownership	1.27	0.63 to 2.56	1.28	0.65 to 2.50
Household income loss during COVID-19	0.39**	**0.18 to 0.85**	0.41**	**0.19 to 0.86**
Partner secondary or higher education	1.11	0.49 to 2.50	1.13	0.51 to 2.51
Partner has other wives	0.96	0.41 to 2.25	1.09	0.47 to 2.54
Participates in decisions on husband’s earnings	0.75	0.33 to 1.73	0.75	0.32 to 1.76
Continuous non-participation 2020–2021	Ref		Ref	
Participation reduced 2020–2021	0.53	0.11 to 2.47	0.48	0.11 to 2.16
Participation increased 2020–2021	0.47	0.13 to 1.69	0.45	0.13 to 1.58
Continuous participation 2020–2021	0.49	0.11 to 2.21	0.45	0.10 to 1.92
More IPV justification county norms (women)	–		0.56	0.22 to 1.43
More IPV justification county norms (men)	–		3.03	0.72 to 12.80
Observations	250		250	

Covariate measures taken at first time point, 2020, unless otherwise noted.

Covariates identified through backwards stepwise regression.

Logistic regression accounting for survey design and weighting.

*p<0.10; **p<0.05; ***p<0.01. Bolding signifies p<0.10

aOR, adjusted OR; IPV, intimate partner violence; Ref, reference.

**Table 5:** Multivariable regression of select indicators on any IPV and IPV trajectories, county-level norms stratified

	Women county norms				Men county norms			
	Less IPV justification counties^†^		More IPV justification counties^‡^		Less IPV justification counties^†^		More IPV justification counties^‡^	
	aOR	95% CI	aOR	95% CI	aOR	95% CI	aOR	95% CI
*Any IPV vs sustained safety*								
Secondary or higher education	0.70*	**0.49 to 1.00**	0.91	0.58 to 1.43	0.66**	**0.4 to 0.97**	0.93	0.62 to 1.41
Lowest wealth	Ref		Ref		Ref		Ref	
Middle wealth	0.66**	**0.45 to 0.98**	0.98	0.58 to 1.63	0.66**	**0.44 to 0.99**	0.93	0.57 to 1.53
Highest wealth	0.69	0.40 to 1.20	0.63	0.32 to 1.26	0.73	0.39 to 1.35	0.59*	**0.33 to 1.06**
Any savings	0.71	0.46 to 1.10	0.70	0.43 to 1.14	0.71	0.44 to 1.15	0.69	0.45 to 1.08
Partner has other wives	2.00**	**1.10 to 3.63**	1.43	0.86 to 2.36	2.10**	**1.15 to 3.85**	1.34	0.81 to 2.21
Participates in decisions on husband’s earnings	0.59***	**0.41 to 0.86**	0.67*	**0.44 to 1.04**	0.63**	**0.43 to 0.94**	0.60**	**0.41 to 0.89**
Observations	1591		902		1421		1072	
*Sustained IPV vs IPV cessation*								
Age 15–29 years	Ref		Ref		Ref		Ref	
Age 30–39 years	2.14	0.75 to 6.13	0.87	0.22 to 3.50	1.92	0.67 to 5.47	0.9	0.24 to 3.34
Age 40–49 years	1.01	0.30 to 3.44	1.42	0.29 to 6.85	0.96	0.27 to 3.37	1.37	0.30 to 6.25
Secondary or higher education	1.04	0.24 to 4.61	0.18**	**0.04 to 0.74**	1.19	0.23 to 6.22	0.17**	**0.04 to 0.66**
Three or more children (vs 0–2)	2.14	0.75 to 6.13	0.87	0.22 to 3.50	1.92	0.67 to 5.47	0.9	0.24 to 3.34
Lowest wealth	Ref		Ref		Ref		Ref	
Middle wealth	2.32	0.81 to 6.66	0.59	0.20 to 1.73	2.37	0.82 to 6.89	0.58	0.20 to 1.72
Highest wealth	0.25*	**0.06 to 1.11**	0.86	0.21 to 3.58	0.29	0.06 to 1.43	0.5	0.14 to 1.86
Any savings	0.45	0.11 to 1.88	1.39	0.45 to 4.33	0.48	0.11 to 2.16	1.39	0.46 to 4.21
Continuous non-work 2020–2021	Ref		Ref		Ref		Ref	
Worked to not work 2020–2021	0.39	0.06 to 2.51	2.76	0.46 to 16.74	0.37	0.06 to 2.44	2.88	0.48 to 17.22
Not worked to work 2020–2021	1.34	0.29 to 6.05	0.95	0.23 to 3.88	1.32	0.28 to 6.34	0.97	0.24 to 3.83
Continuous work 2020–2021	1.03	0.29 to 3.68	1.95	0.50 to 7.60	1.02	0.27 to 3.78	1.99	0.53 to 7.53
Mobile money account ownership	2.67*	**0.87 to 8.21**	0.74	0.27 to 2.01	2.74*	**0.84 to 8.91**	0.78	0.28 to 2.22
Household income loss during COVID-19	0.58	0.20 to 1.72	0.33*	**0.11 to 1.01**	0.56	0.17 to 1.82	0.33*	**0.11 to 1.01**
Partner secondary or higher education	1	0.30 to 3.41	1.45	0.41 to 5.11	0.9	0.24 to 3.37	1.39	0.41 to 4.73
Partner has other wives	1.13	0.25 to 5.02	0.87	0.30 to 2.52	1.02	0.21 to 4.91	0.88	0.30 to 2.58
Participates in decisions on husband’s earnings	0.43	0.12 to 1.59	1.26	0.35 to 4.55	0.4	0.11 to 1.51	1.39	0.38 to 5.03
Continuous non-participation 2020–2021	Ref		Ref		Ref		Ref	
Participation reduced 2020–2021	0.56	0.09 to 3.55	0.17	0.01 to 2.16	0.59	0.09 to 3.70	0.18	0.01 to 2.14
Participation increased 2020–2021	0.44	0.08 to 2.41	0.34	0.04 to 2.97	0.43	0.07 to 2.50	0.34	0.04 to 2.87
Continuous participation 2020–2021	0.39	0.06 to 2.62	0.36	0.03 to 3.71	0.43	0.06 to 2.99	0.34	0.04 to 3.21
Observations	136		114		128		122	

Covariate measures taken at first time point, 2020, unless otherwise noted.

Covariates identified through backwards stepwise regression.

Logistic regression accounting for robust standard error clustering and survey weighting.

*p<0.10; **p<0.05; ***p<0.01. Bolding signifies p<0.10

†Counties with or below weighted average of number of actions (0–5) that DHS participants in respondent’s county say justify physical IPV.

‡Counties above weighted average of number of actions (0–5) that DHS participants in respondent’s county say justify physical IPV.

aOR, adjusted OR; DHS, Demographic and Health Survey; IPV, intimate partner violence; Ref, reference.

The odds of sustained IPV, compared with IPV cessation, were lower among women who reported household income loss due to COVID-19 restrictions (aOR 0.39, 95% CI 0.18 to 0.85), meaning that women with household income loss were more likely to experience IPV cessation (table 4). This association remained when adjusting for county-level IPV justification norms. County-level norm measures were not associated with sustained IPV compared with IPV cessation. In counties with higher normative IPV justification among women and men, women’s secondary education decreased the odds of sustained IPV relative to IPV cessation (aOR 0.18, 95% CI 0.04 to 0.74; and aOR 0.17, 95% CI 0.04 to 0.66) with no such association in counties with less normative IPV justification (table 5). No independent variables or county-level norms measures associated with new IPV compared with sustained safety (results available upon request).

## Discussion

This first prospective study of IPV trajectories among women in Kenya on the heels of the COVID-19 pandemic demonstrates striking consistency in overall IPV prevalence over a 1-year period, indicative of unmet needs for prevention and response. Prevalence of new IPV and IPV cessation across the 1-year period was similar, suggesting frequent ‘in-and-out’ trajectories. The individual and household factors associated with any IPV over the 1-year period are consistent with past research demonstrating IPV risk.^47 48^ A main contribution of the study is the finding that few factors associated with outcomes of *change* in IPV across the 1-year period, suggesting that individual characteristics do not affect change much within a 1-year period. While some research on IPV trajectories has been explored in relation to mental health, childhood abuse and witnessing IPV as a child, there are few relevant studies exploring sociodemographic associations within the sub-Saharan African context with which to compare.^13 49^ The dynamic movement in and out of IPV risk over the course of 1 year demonstrates the value of prospective research on this topic and the need for longer-term follow-up to better understand movement in and out of safety.

The prevalence of help-seeking in this study, around 40% across rounds, is similar to that which was found in the 2022 Kenya DHS for female survivors of physical or sexual violence (42%).^50^ Surprisingly, neither informal nor formal help-seeking in 2020 was associated with IPV cessation in 2021 compared with those with sustained IPV. However, among those who sought formal help in 2021, a greater share had sustained IPV than new IPV; these results suggest that it takes time for survivors to access help and for that help to translate into safety and are consistent with past evidence that survivors may initiate help-seeking following violence severity and/or chronicity.^51 52^ Findings may be further explained by persistent stigma associated with seeking help, institutional mistrust and low-quality survivor services identified through recent qualitative work in Kenya.^53^

A unique contribution of the study is the rich insight into the normative environment that shapes IPV risk, specifically the role of community-level IPV justification norms. First, it is striking that women’s reported norms that endorse IPV justification at the county level were associated with increased IPV risk, while no such associations were observed for men’s reported IPV justification. Further, women generally reported higher justification of IPV than men. This pattern may reflect a ‘coping through tolerance’ model by which women may adopt norms of IPV tolerance as a coping strategy, in a mutually reinforcing model of IPV tolerance and experience, particularly where the cost of separation is high, as it is in Kenya. Available evidence from India suggests that long-term exposure to violence shifts women’s attitudes towards violence, prompting women to normalise and rationalise IPV as a coping mechanism, particularly if no outside option exists.^54 55^ Norms data were derived via the Kenya DHS independently from the IPV experience data, though it is possible that witnessing or experiencing violence increases report of IPV justification.

The second key learning on how the normative environment shapes IPV risk pertains to the interplay of normative and individual factors. Specifically, while factors such as co-wives and education were associated with any contact IPV at either time point, these individual-level factors were less, if at all, associated with IPV in settings where there were stronger norms justifying IPV. In other words, individual-level factors appear to be less relevant to IPV risk in settings where norms justifying IPV are strong. By contrast, as norms shift away from IPV justification, individual-level factors become more relevant. These findings suggest that the social normative context is ‘activating’—demonstrating a complex interplay across the social ecology. Taken together, this current study’s identification of few individual factors associating with 1-year IPV trajectories and the significant role of county-level norms suggests that normative environments are sticky; subsequently, IPV risk may not change much in such a short period. Such evidence highlights a need for future work to contribute to the small but growing evidence base on how associations between individual factors and IPV are influenced by the normative context, such as recent evidence exploring women’s heightened risk of IPV when going against the economic empowerment norm.^56 57^

Study strengths include the prospective study design and the integration of multiple data sources which limits bias. The study has several limitations. While the prospective design is a strength, the follow-up period was relatively limited and may not be sufficiently long enough to capture changes, particularly for partnered women in midlife. The relative lack of identified risk and protective factors for movements in and out of safety may suggest the 1-year period was an insufficient reference period in identifying drivers of change. However, our analysis investigates the nuance of change in this period by exploring all four possible trajectories. Due to survey length limitations, we lack clarity on the nature, intensity and impact of help-seeking, as well as the chronicity of IPV, including that which predated the initial survey round. We seek to address this in part by exploring informal and formal help-seeking separately. It is also possible that the most relevant contextual triggers of relationship safety were not assessed (eg, relationship conflict, economic shifts and more subtle nuances). Our study is limited in measures of relationship dynamics between partners, as well as data on mental health and alcohol use for individuals and their partners that are known IPV risk factors and may serve as more proximate risk drivers.^58 59^ While limited, we do include measures on household decision-making, including pertaining to the partner’s earnings, which provide some insight into couple dynamics. Last, though the DHSs are rigorously validated, injunctive norms are difficult to assess. These measures may have measurement bias, potentially differential between genders.

Taken together, results highlight persistent IPV that threatens the safety and well-being of women and their families and communities. While movement towards safety over a 1-year period for 7% of women is promising, these gains are offset by incident IPV for 8%. To truly understand the impact of recent policy and programmatic advances on risk, the evidence base must move beyond cross-sectional research and increasingly examine risk and protective factors for sustained IPV and IPV cessation, across a longer follow-up period and with deep attention to the normative context as well as relational dynamics. Future studies on IPV trajectories will also benefit from a mixed-methods design that includes a qualitative life history approach to gain a fuller understanding of how individual IPV experience is embedded into the broader socionormative context.

Findings have implications for policy and practice in Kenya and beyond. Given low formal help-seeking and no identified association between help-seeking and IPV cessation, improved infrastructure and norms change to promote help-seeking are needed in Kenya, as recommended by recent work on help-seeking in Kenya.^53^ Results are particularly timely in light of the global attention on Kenya’s femicide epidemic, punctuated by high-profile IPV homicides. Social norms programming is needed: transforming attitudes, beliefs and norms is a pillar of the UN Women RESPECT framework strategy to reduce violence against women and girls, and promising evidence suggests that norms change is possible and can make an impact.^60^ Combined with efforts to reduce barriers to help-seeking with precision, locally informed social norms change interventions can contribute to preventing continued revictimisation of IPV survivors over time.

## Supplementary material

10.1136/bmjgh-2025-021078 online supplemental figure 1

10.1136/bmjgh-2025-021078 online supplemental figure 2

10.1136/bmjgh-2025-021078 online supplemental file 1

## Data Availability

Data are available in a public, open access repository.
