# Supplementary figures and images for: Intimate partner violence trajectories over a 1-year period in a population-based cohort of women in Kenya: associations with individual and community normative factors
Source: BMJ Glob Health. 2025 Dec 25;10(12):e021078. doi: 10.1136/bmjgh-2025-021078 (PMC12742071; doi:10.1136/bmjgh-2025-021078)

Severe IPV  
6.1% (n=151)

Less Severe IPV  
5.3% (n=132)

No Contact IPV  
88.7% (n=2,215)

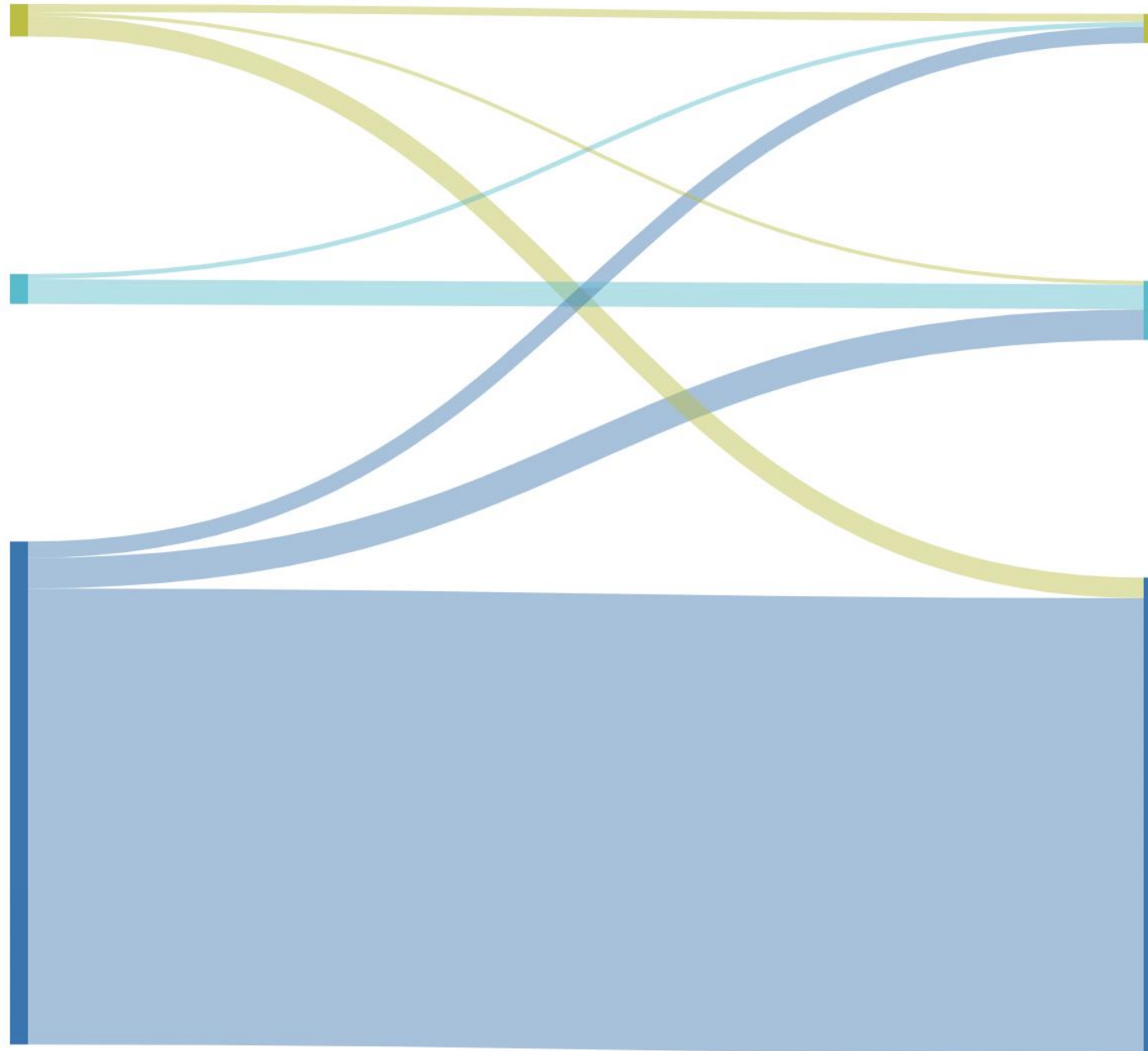

Severe IPV  
5.4% (n=134)

Less Severe IPV  
7.1% (n=178)

No Contact IPV  
87.5% (n=2,187)

Supplement: online supplemental figure 1 [file bmjgh-10-12-s001.pdf]

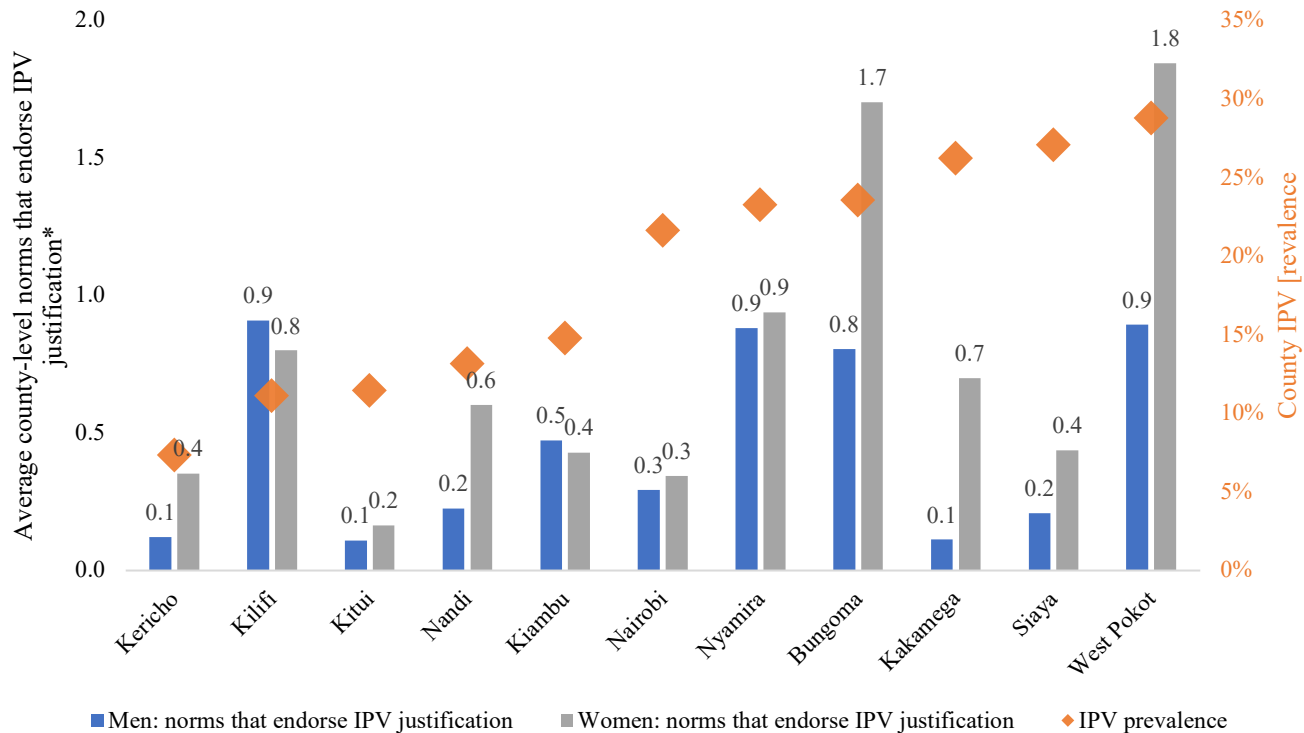

Supplement: online supplemental figure 2 [file bmjgh-10-12-s002.pdf]
