# Supplementary material for: Intimate partner violence trajectories over a 1-year period in a population-based cohort of women in Kenya: associations with individual and community normative factors
Source: BMJ Glob Health. 2025 Dec 25;10(12):e021078. doi: 10.1136/bmjgh-2025-021078 (PMC12742071; doi:10.1136/bmjgh-2025-021078)
Supplement: online supplemental file 1 [file bmjgh-10-12-s003.docx]

**Supplemental Figure 1: Trajectories of past-year contact IPV by IPV severity from 2020 to 2021 (n=2,499, weighted)**

**Supplemental Figure 2: Figure 3: Average county-level (n=11 counties) norms that endorse IPV justification from DHS data with corresponding contact IPV prevalence at either time point from PMA data, weighted, by gender**
